# Cationic polyamines inhibit anthrax lethal factor protease

**DOI:** 10.1186/1471-2210-6-8

**Published:** 2006-06-08

**Authors:** Mark Evan Goldman, Lynne Cregar, Dominique Nguyen, Ondrej Simo, Sean O'Malley, Tom Humphreys

**Affiliations:** 1 Hawaii Biotech, Inc., 99-193 Aiea Heights Dr., Aiea, HI 96701, USA

## Abstract

**Background:**

Anthrax is a human disease that results from infection by the bacteria, *Bacillus anthracis *and has recently been used as a bioterrorist agent. Historically, this disease was associated with *Bacillus *spore exposure from wool or animal carcasses. While current vaccine approaches (targeted against the protective antigen) are effective for prophylaxis, multiple doses must be injected. Common antibiotics that block the germination process are effective but must be administered early in the infection cycle. In addition, new therapeutics are needed to specifically target the proteolytic activity of lethal factor (LF) associated with this bacterial infection.

**Results:**

Using a fluorescence-based assay to identify and characterize inhibitors of anthrax lethal factor protease activity, we identified several chemically-distinct classes of inhibitory molecules including polyamines, aminoglycosides and cationic peptides. In these studies, spermine was demonstrated for the first time to inhibit anthrax LF with a K_i _value of 0.9 ± 0.09 μM (mean ± SEM; n = 3). Additional linear polyamines were also active as LF inhibitors with lower potencies.

**Conclusion:**

Based upon the studies reported herein, we chose linear polyamines related to spermine as potential lead optimization candidates and additional testing in cell-based models where cell penetration could be studied. During our screening process, we reproducibly demonstrated that the potencies of certain compounds, including neomycin but not neamine or spermine, were different depending upon the presence or absence of nucleic acids. Differential sensitivity to the presence/absence of nucleic acids may be an additional point to consider when comparing various classes of active compounds for lead optimization.

## Background

Anthrax is a disease of animals including humans and results from infection by *Bacillus (B.) anthracis *[1-3]. Anthrax spores reside in soil samples worldwide and are resistant to environmental insults such as temperature, moisture and UV irradiation. Spores enter host animals via inhalation, epidermal or gastrointestinal routes with respiratory route being the most fatal. Once inside hosts, the spores germinate and secrete three toxin components, called lethal factor (LF), edema factor (EF) and protective antigen (PA), encoded by the pXO1 plasmid. LF, a metalloprotease, plus PA is termed lethal toxin and EF plus PA is termed edema toxin [3]. The combination of bacteremia and release of the protein toxins leads to sepsis, pulmonary edema and other fatal effects [1-5].

PA is responsible for translocating the two other gene products, LF and EF, into the cytosol of susceptible cells [6-9]. The precursor form of PA (PA83) binds to ubiquitous cell surface receptors including von Willebrand factor, tumor endothelial marker 8 (TEM8) and capillary morphogenesis protein 2 [10,11]. PA83 is cleaved by furin as well as by serum proteases [12-14]. The active form of PA (PA63) then heptamerizes and binds with a high affinity to LF or EF [3]. The complex of PA with LF or EF forms a channel to allow LF/EF to translocate from the endosome to the cytosol where the toxic effects associated with LF are manifest [3]. Cationic peptides that inhibit furin-mediated activation of PA83 to PA63 are also effective in blocking lethal toxin cytotoxicity [13,16,17].

EF is a calmodulin-dependent adenylate cyclase and thus elevates intracellular cAMP levels of intoxicated cells [18]. As a result of this mechanism, EF causes the additional pathological effects in the host although it is less virulent than LF. Recent studies have demonstrated that adefovir diphosphate is a potent inhibitor of anthrax EF, *in vitro *[19].

Anthrax LF is a representative member of the zinc-dependent endopeptidases family as demonstrated by the presence of the HEXXH zinc-binding consensus sequence [3,15]. LF, an 89 kD protein, is one of the main virulence factors of anthrax [3,15]. LF contains numerous anionic sites both within the active site and at distant sites [20-22].

Macrophages are target cells of LF toxicity in animal model systems. Exposure of murine macrophages to lethal toxin resulted in rapid loss of cell viability [3,23,24]. Conversely, mice depleted of macrophages were not sensitive to lethal toxin [1-3]. The mechanism of lethality has been attributed to release of cytokines or apoptosis as well as other mechanisms but is highly dependent upon the LF concentration [1-3,5,26-29].

The mitogen activated protein kinase/extracellular signal-regulated protein kinase (MAPK/ERK) pathway is a major regulator for communication of extracellular signals to the nucleus and is involved in cellular adaptations to the environment [30,31]. In the cytosol, LF cleaves members of the mitogen activated protein kinase kinase (MAPKK) family in the N-terminal region including MAPKK family members 1–3 [31,32]. The reduced levels of MAPKKs then prevent p38 kinase-mediated activation of immune mechanisms *B. anthracis *to evade host immunological mechanisms. Recently published studies have demonstrated that small molecules can inhibit LF activity and subsequently block LF-mediated cytotoxicity [21,29,33-36].

At present, the only mechanism to fatally "intoxicate" cells with lethal factor is via host infection with *B. anthracis *spores that germinate in lymphatic tissues and secrete their toxin components. None of the three individual gene products of pXO1 are toxic *in vivo *[37]. Inhibitors of other proteases such as angiotensin converting enzyme and HIV-1/HIV-2 proteases are effective and highly specific drugs for the treatment of chronic diseases [38] and therefore suggest a logical strategy for identifying anthrax lethal factor inhibitors. Based upon the demonstration of the anionic rich regions of LF [21,22,29], we chose chemical libraries that included cationic compounds to test for LF inhibition. These studies were directed at identification of compounds that selectively inhibited LF both at the enzyme level then evaluation of their effects in cell culture based assays. An ideal therapeutic would penetrate susceptible cells and be effective protease inhibitors in "post-exposure" models of treatment.

## Results

### Substrate kinetics

Using MAPKKide™ as substrate, velocity vs. substrate curves were analyzed by DYNAFIT, a nonlinear fitting program [39]. The results of the forward progress curves demonstrated a clear substrate inhibition process as previously shown with a different substrate [20]. The K_m _and K_i _values for MAPKKide™ were calculated to be 8.6 ± 1.5 μM and 85 ± 17 μM, respectively. These data suggest that multiple inhibitory mechanisms may be available as sites for binding of LF inhibitors (Kuzmic *et al*., submitted).

### Endogenous polyamines inhibit Lethal Factor enzyme activity

**Figure 1:**
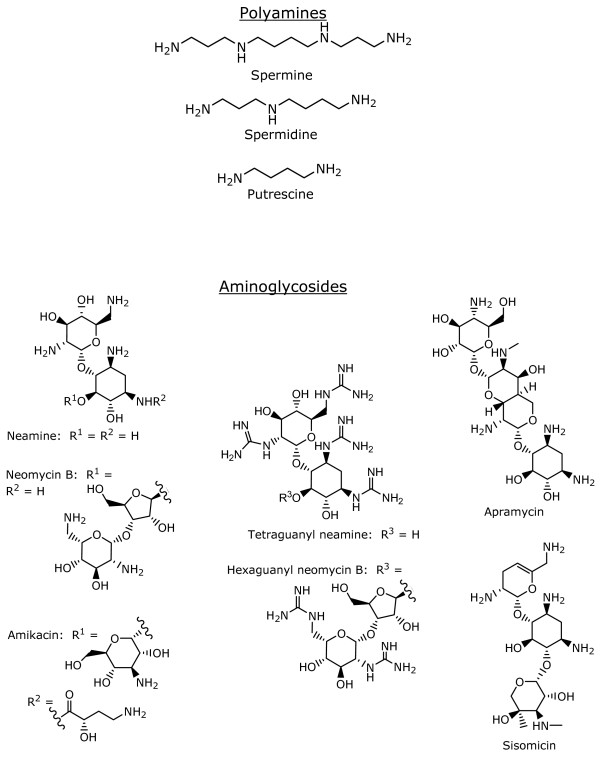
Chemical structures of compounds used in this study.

**Figure 2:**
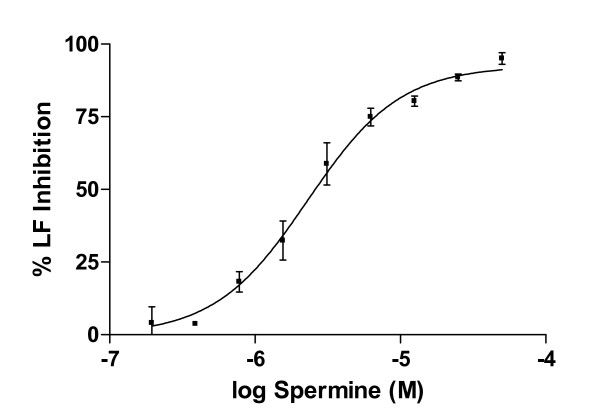
Spermine inhibits anthrax lethal factor protease in a concentration-dependent manner. These results (mean ± SEM) are averaged from 3 separate experiments.

Based upon the presence of anionic sites on LF, we hypothesized that cationic compounds, including members of known drug-like chemical families, might inhibit LF enzyme activity. An initial focused library of commercially available cationic compounds (n~100 compounds) from numerous chemical classes was assembled and tested at a concentration of approximately 10 μg/ml in the LF enzyme inhibition assay. One of these compoundsspermine, was found to inhibit anthrax protease activity in a concentration-dependent manner with a K_i _value of 0.9 ± 0.09 μM (mean ± SEM; Figure 1; Figure 2; Table 1). In contrast to the potent inhibition of anthrax LF protease enzyme activity, this compound was >40-fold weaker as a botulinum protease inhibitor (K_i _value = 46 ± 6 μM) and much less active on mammalian proteases including trypsin, cathepsin B and cathepsin D (IC_50 _values > 500 μM). Several other endogenous polyamines, including spermidine, and ornithine, were evaluated for activity as LF inhibitors; these endogenous compounds were weaker than spermine but were still concentration-dependent inhibitors (Table 1).

**Table 1:** Endogenous polyamine and aminoglycoside-mediated inhibition of anthrax lethal factor activity-comparison with other proteases-(Mean ± SEM Kiapp
 MathType@MTEF@5@5@+=feaafiart1ev1aaatCvAUfKttLearuWrP9MDH5MBPbIqV92AaeXatLxBI9gBaebbnrfifHhDYfgasaacH8akY=wiFfYdH8Gipec8Eeeu0xXdbba9frFj0=OqFfea0dXdd9vqai=hGuQ8kuc9pgc9s8qqaq=dirpe0xb9q8qiLsFr0=vr0=vr0dc8meaabaqaciaacaGaaeqabaqabeGadaaakeaacqqGlbWsdaqhaaWcbaGaemyAaKgabaGaemyyaeMaemiCaaNaemiCaahaaaaa@336E@ or IC_50 _values)

	LF	Bot	MMP-9	Furin
Name	Kiapp MathType@MTEF@5@5@+=feaafiart1ev1aaatCvAUfKttLearuWrP9MDH5MBPbIqV92AaeXatLxBI9gBaebbnrfifHhDYfgasaacH8akY=wiFfYdH8Gipec8Eeeu0xXdbba9frFj0=OqFfea0dXdd9vqai=hGuQ8kuc9pgc9s8qqaq=dirpe0xb9q8qiLsFr0=vr0=vr0dc8meaabaqaciaacaGaaeqabaqabeGadaaakeaacqqGlbWsdaqhaaWcbaGaemyAaKgabaGaemyyaeMaemiCaaNaemiCaahaaaaa@336E@ μM	IC_50 _μM	Kiapp MathType@MTEF@5@5@+=feaafiart1ev1aaatCvAUfKttLearuWrP9MDH5MBPbIqV92AaeXatLxBI9gBaebbnrfifHhDYfgasaacH8akY=wiFfYdH8Gipec8Eeeu0xXdbba9frFj0=OqFfea0dXdd9vqai=hGuQ8kuc9pgc9s8qqaq=dirpe0xb9q8qiLsFr0=vr0=vr0dc8meaabaqaciaacaGaaeqabaqabeGadaaakeaacqqGlbWsdaqhaaWcbaGaemyAaKgabaGaemyyaeMaemiCaaNaemiCaahaaaaa@336E@ μM	Kiapp MathType@MTEF@5@5@+=feaafiart1ev1aaatCvAUfKttLearuWrP9MDH5MBPbIqV92AaeXatLxBI9gBaebbnrfifHhDYfgasaacH8akY=wiFfYdH8Gipec8Eeeu0xXdbba9frFj0=OqFfea0dXdd9vqai=hGuQ8kuc9pgc9s8qqaq=dirpe0xb9q8qiLsFr0=vr0=vr0dc8meaabaqaciaacaGaaeqabaqabeGadaaakeaacqqGlbWsdaqhaaWcbaGaemyAaKgabaGaemyyaeMaemiCaaNaemiCaahaaaaa@336E@ μM
Neomycin B tris-sulfate	0.71 ± 0.04	92.5	>300	N/T
Sisomicin Sulfate	1.8	>300	>300	N/T
Spermine, diphosphate salt	0.9 ± 0.09	57	>300	N/T
Amikacin	23.3	>300	>300	N/T
Neamine (free base)	31.1 ± 5.6	175	>300	N/T
Spermidine	>100	76	N/T	N/T
Apramycin	111	>300	>300	N/T
Putrescine (1,4-diaminobutane)	>300	>300	>300	N/T
Ac-CRATKML-N	>300	3.5	N/T	N/T
GM 6001	7.2 ± 1.76	>300	0.002 ± 0.001	N/T
H-RRRRRR-OH	0.24	N/T*	N/T	0.06
Ac-RRRRRR-OH	0.29 ± 0.04	N/T	N/T	0.05
Ac-RRRRRR-NH2	0.12	N/T	N/T	N/T
H-(D-Arg)-(D-Arg)-(D-Arg)-(D-Arg)-(D-Arg)-(D-Arg)-NH2	0.04 ± 0.02	N/T	N/T	0.06
H-R(NO2)R(NO2)	>300	N/T	N/T	N/T
Neomycin B hexaguanyl hexatrifluoroacetate salt	0.03	14	>170	1.5
Tetraguanyl neamine, free base	0.3	N/T	N/T	N/T

*N/T = not tested

### Aminoglycosides inhibit LF enzyme activity

Aminoglycoside antibiotics bind to the polyamine class of glutamate receptors by a mechanism unrelated to antibiotic activity [40-42]. We therefore evaluated a series of commercially-available aminoglycosides (n = 31) to determine their potencies as LF inhibitors. The results demonstrated that some members of this chemical class were potent inhibitors of anthrax LF cleavage of the substrate (Table 1). Both natural aminoglycosides and synthetic aminoglycosides (Table 1) were active. Of these, neomycin was the most potent aminoglycoside with a K_i _value of 0.3 ± 0.1 μM. Based upon chemical size, we chose to focus on neamine and related compounds (n = 20 neamine derivatives).

### Exogenous nucleic acids alter LF inhibition by neomycin

**Figure 3:**
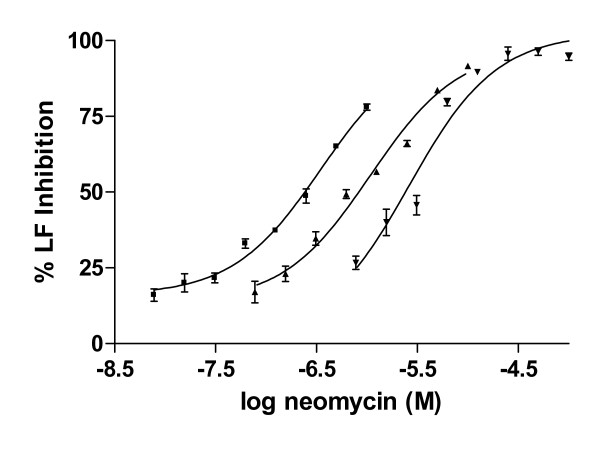
Influence of nucleic acids on concentration-dependent LF inhibition. Anthrax lethal factor activity was measured in the absence of DNA (■) as well as in the presence of salmon sperm DNA at 4 μg/ml (▲) and 8 μg/ml (▼), performed in triplicate.

Since aminoglycosides are known to bind to nucleic acids [43-45] we evaluated key compounds in the absence of DNA (standard assay) and in the presence of a variety of nucleic acids. At concentrations <10 μg/ml, nucleic acids did not affect LF enzyme activity. As shown in Fig 3, the potency of neomycin was greater in the absence of DNA compared to in the presence of salmon testes DNA (4 and 8 μg/ml). The higher concentration of DNA caused a ~10-fold right shift in potency of neomycin. In contrast, the concentration-dependent inhibitory activities of neamine or spermine were unaffected by DNA or RNA (specifically human placental DNA, type III RNA, polyA-polyU; results not shown).

### Cationic peptides inhibit furin enzyme activity

Cross-inhibition of LF and furin has been demonstrated for polyarginine based inhibitors [46]. We therefore examined the ability of our panel of LF inhibitors to inhibit furin in an *in vitro *substrate cleavage assay. As expected, several polyarginine derivatives inhibited furin activity (Table 1). None of the remaining compounds interfered with furin activity at concentrations up to 100 μM.

## Discussion

In the initial phase of this study, we sought to identify compounds that selectively inhibited anthrax lethal factor enzyme activity. Such compounds were hypothesized to be potential lead molecules for optimization as drugs to treat *B. anthracis *infection. Since inhibitors of this protease were not known at the time, we chose to screen structurally diverse collections of individual compounds (a "library" consisting of ~500 compounds in several chemical classes) as one approach towards lead identification. We included simple linear cationic polyamines (n = 17) in the screening library with the hypothesis that they might bind to anionic sites on LF and thus block substrate cleavage. The data presented in this study show that spermine (a simple linear polyamine) is a concentration-dependent, sub-micromolar inhibitor of LF with reduced inhibitory potencies (termed selectivity) versus other bacterial and mammalian proteases. Polyamine analogs of spermine, including spermidine and ornithine were less active than spermine but still displayed concentration-dependent inhibitory effects as LF inhibitors.

Based upon literature demonstrating that both polyamines and aminoglycoside antibiotics bind to the N-methyl-D-aspartate receptor [47,48], we also evaluated aminoglycoside antibiotics for LF inhibition. In our independent studies reported here and identified by other laboratories [49-51] we found that gentamicin inhibited LF enzyme activity without inhibiting other proteases from bacterial and mammalian sources. We then showed that other compounds were more potent LF inhibitors than gentamicin. To further validate the mechanism, we tested cationic peptides (n~5) such as D- and L-hexaarginine as well as non-peptidyl cationic polymers including poly-L-arginine and poly-L-lysine (molecular weight ranges = 5,000–15,000); the larger cationic polymers (both peptidyl and non-peptidyl) were more potent inhibitors. While these large molecules will not be drug leads, they validated the mechanistic hypotheses of LF inhibition. Based upon these data, we concluded that neamine possessed the most relevant combination of drug-like properties and it was used as a scaffold for designing more potent and cell permeable analogs [52].

Aminoglycosides are effective antibiotics for the treatment of Gram-positive and Gram-negative infections as well as certain mycobacterial infections [53,54]. Their use, however, is limited by lack of oral absorption and toxicity at high doses including both ototoxicity and nephrotoxicity [55]. Because of such toxicities, intravenous use of aminoglycosides in large and diverse age/health populations would pose a significant risk if used as prophylactic agents. Orally active/non-toxic compounds are still needed for protection against bioterrorist threats based on *B. anthracis *and its toxins.

High affinity polyamine interactions with nucleic acids are well known in both cell-free and cellular systems [56-60]. In anticancer studies, for example, exogenous polyamines are cytotoxic by depleting endogenous polyamine levels through feedback inhibition mechanisms [59]. The cellular uptake of linear polyamines is well-recognized and numerous transporters have been shown to modulate polyamine levels within cells and organelles [60 and references therein].

We also sought to determine if the presence of DNA or RNA in the MAPKK cleavage assays would affect polyamine inhibition of LF activity. First, we demonstrated that nucleic acids did not inhibit LF activity at concentrations below those known to be present in human blood (< 80 μg/ml; Promega tech bulletin). Subsequently, we have also demonstrated that the presence of these nucleic acids prevent certain compounds such as neomycin but not neamine from inhibiting LF activity. One hypothesis we have considered is the role of size of the inhibitory molecules; smaller compounds such as spermine and neamine did not bind nucleic acids whereas larger molecules that inhibited LF (neomycin) were rendered less potent in the presence of 4–8 μg/ml of DNA or RNA. This is a unique pharmacological discovery first demonstrated in this effort.

All three chemical classes (linear polyamines, aminoglycosides and peptides) are highly charged molecules; they were not expected to be active in cell models of anthrax lethal factor cytotoxicity. This result was confirmed in our initial LF cytotoxicity studies with RAW 264.7 macrophage cells as all compounds were not active up to the highest concentration tested (500 μM). Recent studies [50], however, have demonstrated that aminoglycosides at "seemingly physiological conditions" inhibit LF and exhibit antibiotic activity against *B. anthracis*. In addition, linear polyamines enter cells by multiple mechanisms including active transport [48,59,60]. These different results highlight the need for continued research in this area including longer compound exposure periods.

Inhibition of the proprotein convertase, furin, by cationic peptides was expected since cationic hexapeptides and nonapeptides have been demonstrated to inhibit furin activity [12,13,46]. In contrast, however, the charged, nonpeptidyl compounds were inactive as furin inhibitors at concentrations up to 10 μM. It is likely that other small molecule cations will inhibit furin processing. Such compounds may be effective in a broad spectrum of diseases where furin cleavage of proteins play a role, such as Alzheimer's disease, viral infections and bacterial infections [13].

## Conclusion

Taken together, the enzymologic and pharmacologic results presented in this study demonstrate the role of anionic sites on anthrax lethal factor that specifically bind cationic compounds from different chemical classes. Ultimately multi-drug and multi-dose combinations of antibiotics that suppress production of anthrax spores plus edema factor and LF inhibitors that target intracellular toxins will be employed to treat people exposed to anthrax gene products. Recently, an antiviral agent called adefovir was found to inhibit edema factor adenylate cyclase activity [19]. Combinations of edema factor and lethal factor inhibitors along with Gram-positive antibiotics could be utilized to treat people exposed to anthrax or its gene products. As a spin-off of these efforts, researchers may want to determine if the presence/absence of nucleic acids affects the potency of selected compounds.

## Methods

### Materials

Aminoglycosides, nucleic acids and endogenous polyamines used in this study were purchased from Sigma (St. Louis MO), and Calbiochem (San Diego, CA). Synthetic polyamines were purchased from Mixture Sciences Inc. (La Jolla, CA). Tetrapeptides were synthesized by Synpep, Inc. (Dublin, CA).

### Lethal Factor protease assay

Lethal Factor (20 nM final concentration) and MAPKK substrate (MAPKKide^® ^12.5 μM, final concentration) were purchased from List Biological Laboratories, Campbell, CA and used according to the fluorescence resonance energy transfer (FRET) method. The assay final volume was 50 μl (Fisher #3694 96-well half area plates) consisting of 5 μl inhibitor/test sample/buffer, 25 μl buffer (20 mM Hepes, pH 7.4), 10 μl enzyme and 10 μl substrate. Test sample, buffer and enzyme were incubated briefly at room temperature. Upon addition of substrate, the reaction was linear for 15 min at room temperature. Fluorescence intensity was determined in the kinetic mode (Ex: 320 nm, Em: 420 nm; 6-minute read time; Molecular Devices Gemini fluorescence plate reader) and data was captured by SoftMax Pro (Molecular Devices, Sunnyvale, CA). Analysis of resulting kinetic data was carried out using DYNAFIT and Batch Ki (Biokin, Ltd., Pullman, WA) then plotted with Prism (Graphpad, San Diego, CA).

### Other protease assays

Additional FRET-based substrate cleavage assays were established to monitor specificity of LF inhibitors. The botulinum neurotoxin/A (BoNT/A) assay was carried out in a similar manner as the lethal factor assay (above) except the buffer was composed of 30 mM Hepes, pH 7.3, 5 mM dithiothreitol, 0.25 mM ZnCl_2_, and 1 mg/mL bovine serum albumin (BSA). The substrate for the BoNT/A was SNAPtide^® ^(12.5 μM, final concentration) purchased from List Biological Laboratories (Campbell, CA). BoNT/A enzyme was obtained from the University of Wisconsin.

Furin inhibition was quantified [12] by measuring the hydrolysis of the fluorogenic furin substrate Pyr-RTKR-CMK (Peptide Institute, Osaka, Japan). Assays were performed in 96-well plates using 100 μM substrate, and a serial dilution of inhibitors. The initial velocity (V_o_) of the 200 μl reactions was quantified using a Spectramax Gemini XS microplate reader. The IC_50 _of each inhibitor was calculated by plotting V_0 _versus log [I] and performing nonlinear regression. K_i(app) _was calculated from the IC_50 _values using the equation K_i _= IC_50_/1+([S]/K_m_.

### Cell-based cytotoxicity assay

RAW 264.7 murine macrophage cells (ATCC, Manassas, VA) were grown in the presence of Dulbecco's modified Eagles medium containing 10% FBS to 70% confluency (approximately 50,000 cells/well for 24 hours) in standard Corning 96-well cell-culture grade polystyrene plates (Corning, NY). Test compounds at various concentrations (concentration-response) and appropriate vehicles were added to the medium and preincubation was continued for 60 min. At the end of this period, PA (250 ng/ml) and LF (250 ng/ml) were added sequentially to each well. After 2 hours, 3-[4,5-dimethylthiazol-2-yl]-2,5-diphenytetrazolium bromide (MTT; 0.5 mg/ml final) was added and the incubation was continued for an additional 2 hours. The supernatant fluid was removed from each well and the remaining pigment was dissolved in 100 μl of 0.5% (w/v) sodium dodecyl sulfate, 40 mM HCl in 90% (v/v) 2-propanol. Absorbance was read at 570 nm and % viability was determined as a function of control wells.

## Authors' contributions

**M.E.G. **(principal investigator, chemical library composition, data evaluation, manuscript preparation), **L.C. **(execution of biochemical experiments and data analyses), **D.N. **(establishment of cell assays and execution of cell assays), **O.S. **(synthesis of compound in Table 1), **S.O. **(synthesis of compound in Table 1) and **T.H. **(target conception; critical intellectual discussion with principal investigator and manuscript evaluation/critique)
